# The fusion of keratinized epithelium, an indication of early implant placement in the aesthetic area: an animal study

**DOI:** 10.1186/s12903-023-03755-9

**Published:** 2023-12-19

**Authors:** Chengyan Ren, Weihui Chen, Jiangping Chen, Chuanqing Mao, Caiyu Liao, Jianan Liu

**Affiliations:** 1 Department of Oral and Maxillofacial Surgery, Fujian Medical University Union Hospital, Fuzhou, China; 2 Fujian Key Laboratory of Oral Diseases & Stomatological Key Lab of Fujian College and University, School and Hospital of Stomatology, Fujian Medical University, Fuzhou, China; 3 Department of Oral and Cranio-maxillofacial Surgery, Shanghai Ninth People’s Hospital, College of Stomatology, Shanghai Jiao Tong University School of Medicine, Shanghai, China

**Keywords:** Early implant placement, Indication, Provisional matrix, Osseointegration

## Abstract

**Background:**

In the period of the early implant placement, the socket is mainly occupied by provisional matrix (PM). Keratinized epithelium (KE) is critical for primary wound closure. Although both KE and PM are important, the detailed relationship among migrating KE, PM formation and indication of the early implant placement is still unclear.

**Objective:**

This research aimed to locate a healing stage of KE with highest osteogenic PM formation after tooth extraction, which could be treated as the optimal time point for early implant placement.

**Material and methods:**

Mice were sacrificed on days 1, 2, 3, 4 and 6 after incisor extraction. Clinical, histological, and immunohistochemical evaluations of the extraction sockets were performed, and statistical analyses were conducted. We then inserted implants into the PM with the greatest bioactivity and observed its osseointegration pattern for 3, 10, 17 and 30 days.

**Result:**

When KE fusion was reached, sockets were dominated by PM with the greatest expression of osteocalcin (OC, *P* < 0.05) and high levels of CD34 and Runx2. OC and Runx2 expression were positively correlated with KE coverage (*P* < 0.05). When the implant was inserted at 4 days’ healing, the PM maintained its osteogenic ability, and osseointegration proceeded perfectly.

**Conclusion:**

The migration of KE was correlated with the formation of highly osteogenic and angiogenic PM. And the fusion of KE could be treated as an indication for early implant placement.

**Supplementary Information:**

The online version contains supplementary material available at 10.1186/s12903-023-03755-9.

## Introduction

When implant treatment was first introduced for patients in the 1980s, it became an accepted and dependable treatment for missing teeth [1, 2]. The early implant placement can shorten the treatment time accompanying with good and stable aesthetic outcome, which has aroused great interest among dentists [3–5].

Early implant placement consists of Type II implants, placed after 4–8 weeks of healing, and Type III implants, placed after 8–12 weeks of healing. Both types have shown their advantages in many clinical practices [1, 4–8]. In the traditional concept, the opportunity for implant placement largely depends on histologic changes and healing time following tooth extraction [8].

In the first 6–8 weeks of healing, the extraction socket is full of PM containing mesenchymal stem cells, fibrous matrix and rich blood vessels, which lays the foundation for osteogenesis. Additionally, a great variety of histological and bioactive stages have been found in human during similar healing times [9]. Therefore, the bioactive stage of extraction sockets could not be detected according to the healing time alone. In dogs’ research, Luo et al. found that PM extracted from sockets had great osteogenic ability and could be treated as an autograft in bone defects [10]. However, tissue derived from dog’s sockets in earlier healing time consist of granulation tissue (GT) and clots, which showed almost no osteogenic ability when applied in bone defect. According to the results of the article above, the structure and bioactivity of early healing tissue located in sockets could influence the capability for osteogenesis directly [11]. It is reasonable to speculate that the biological activity of the alveolar socket and tissues within it, which directly contacted with implant surface in Type II and Type III implant, would affect the osseointegration process. Hence, the indication for early implant placement can be viewed from a histological perspective as the identification of a bioactive early healing phase.

There is a close relationship between the timing of epithelial coverage and the dominance of PM in the socket [9, 12]. According to some authors, KE might act as a barrier that protects the apical area of sockets from bacteria, causing GT to be replaced and PM to form [13]. Additionally, increased KE width contributes to wound closure after guided bone regeneration (GBR) procedures, which were usually applied with early implant placement [6, 14]. KE appears to play an important role in PM formation and early implant placement. To date, no prior research has examined the relationship between KE coverage, PM formation and the indication of the early implant placement.

The animal model of mice’s incisor extraction sockets has been widely used in studies of alveolitis [15, 16], as this model, with a single root, facilitates observation. Additionally, this model is suitable to obtain a pre-experimental foundation for large animal or clinical studies. According to previous research, new bone formation occurs at 7 days after tooth extraction, which suggests that if we want to study the healing process of PM, the healing time should not exceed 7 days [17].

This study tries to find a healing stage of KE when the PM form in the alveolar sockets with highest osteogenic activity. Besides, whether PM contacted with implants directly could further contributed to ossteointegration is also within the scope of our research.

## Materials and methods

### Animals

Six-week-old male ICR mice weighing 30–35 g were used in this study. In the study period, the mice, housed in a pathogen-free barrier environment, received standard solid food and sterile water, except in the first 3 hours after tooth extraction, when the food was ground. Mice were randomly divided into 5 groups, each comprising 6 mice (1, 2, 3, 4 and 6 days after upper incisor extraction). In implantation procedure, total 16 mice were divided into 4 groups.

All animal experiments was approved by the Ethics Committee of Fujian Medical University and was in accordance with the ARRIVE guidelines (No. FJMU IACUC 2021–0350).

### Surgical procedure

Mice were anesthetized by an intraperitoneal administration of 40 mg/kg 2% pelltobarbitalum natricum. All surgeries were performed under standard sterile conditions with 2.5× loupe magnification. The animal was placed on a surgical table, and a #11 surgical razor blade was used to separate the soft tissue around the left incisor and promote incisor element movement. Then, the tooth was removed using clinical tweezers. After extraction, the soft tissue in the socket, such as periodontal ligament, was cleaned as much as possible, and the completeness of the incisor was checked. All surgical procedures were performed by a single calibrated surgeon. Incomplete teeth or fractured bone, especially bone of the distal or buccal bone walls, were not observed in any mouse. At the end of the experiments, mice were anesthetized at 1 d, 2 d, 3 d, 4 d and 6 d after tooth extraction for clinical observation of soft tissue healing under a stereoscopic microscope. According to the result of first part, the socket with 4 days’ healing after tooth extraction, an implant was inserted into to simulate early implant placement. After the mice were anesthetized, a titanium implant (Ti-Al-V, 0.6-mm diameter titanium dentine pin; STABILOK) was inserted as described in a previous study, but no primary stability could be obtained [18]. Mice were sacrificed at 3d, 10d, 17d and 30d after implantation.

### Tissue collection, histomorphometric analysis and zone separation

**Fig. 1:**
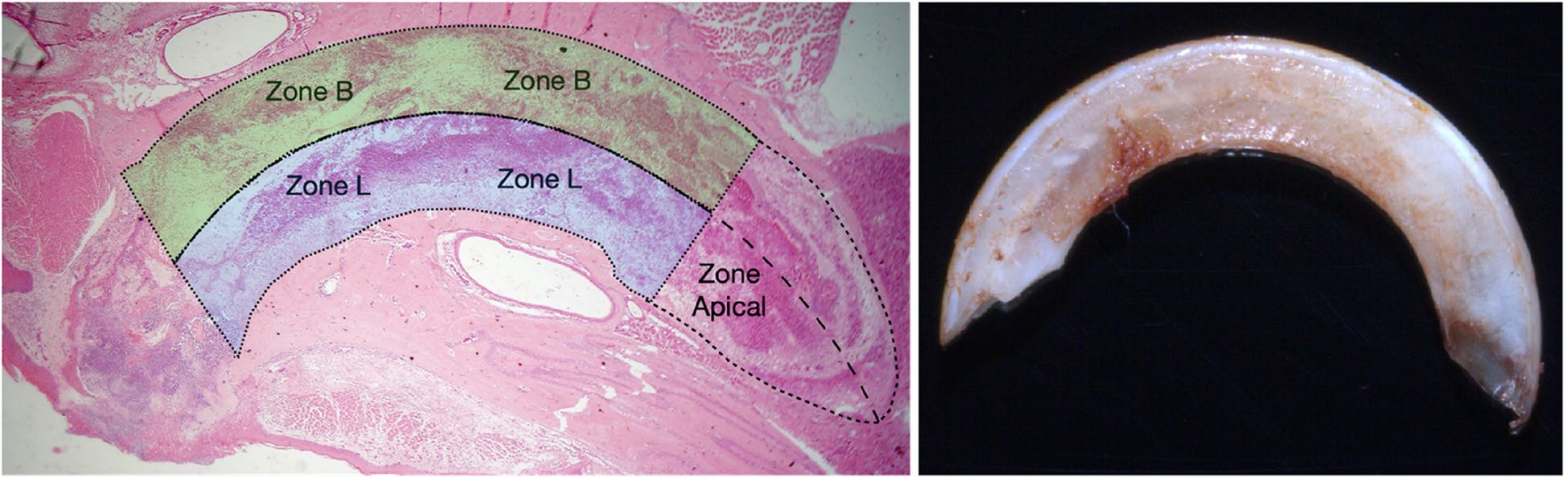
AOI of sockets with HE staining and image of the upper incisor. Blue, Zone L; green, Zone B. The upper incisor was extracted without any fracture

Extraction sockets were collected and fixed with 4% paraformaldehyde for 36 hours. Then, samples were decalcified for 2 weeks in 20% EDTA–glycerol solution. Serial sections with a thickness of 4 μm were prepared for hematoxylin–eosin staining (Solarbio, G1120), masson staining (Maixin, MST-8003), tartrate-resistant acid phosphatase (TRAP, Sigma, 387A) and future experiments. The sections were divided into three parts along the long axis. The area of interest (AOI), including the coronal two-thirds of the socket, was identified. The coronal and middle parts of the tooth were divided into the following regions along the long axis: (1) the buccal zone (Zone B), which was located between the string and buccal or facial wall, and (2) the lingual zone (Zone L), which was located in the region between the axis line and lingual wall (Fig. 1). Before further histological analysis, each sample was checked first whether the nasal bone remained complete and whether any dental pulp or fractured dentin remained. No samples were excluded.

### Immunohistochemistry and evaluation

The primary antibodies for immunohistochemistry (IHC) were Runx2 (ab192256, 1:500), osteocalcin (OC, 1:200) and CD34 (ab81289, 1:1000). ImageJ was used to detect the expression of OC. The integrated optical density (IOD) and average optical density (AOD, average optical density = IOD/area) were calculated.

QuPath was used to count the number of cells stained by Runx2 and newly formed vessels stained by CD34. The number of multinuclear osteoclasts at the crest of the buccal wall was counted.

### Statistical analysis

Differences among all groups were statistically analyzed by one-way analysis of variance (ANOVA) followed by Turkey multiple comparison test. The unpaired Student’s t test was used where applicable for comparisons between any two groups. The relationship between KE and biochemical markers was analyzed by Pearson’s correlation coefficients. If the data in groups did not conform to a normal distribution, the Mann–Whitney and Kruskal–Wallis tests were used. Spearman correlation coefficients were used in the correlation analysis. Statistical significance was defined as 𝑃 < 0.05, and all data were tested with GraphPad Prism 8.0 software.

## Results

### Histological analysis of the early healing pattern

**Fig. 2:**
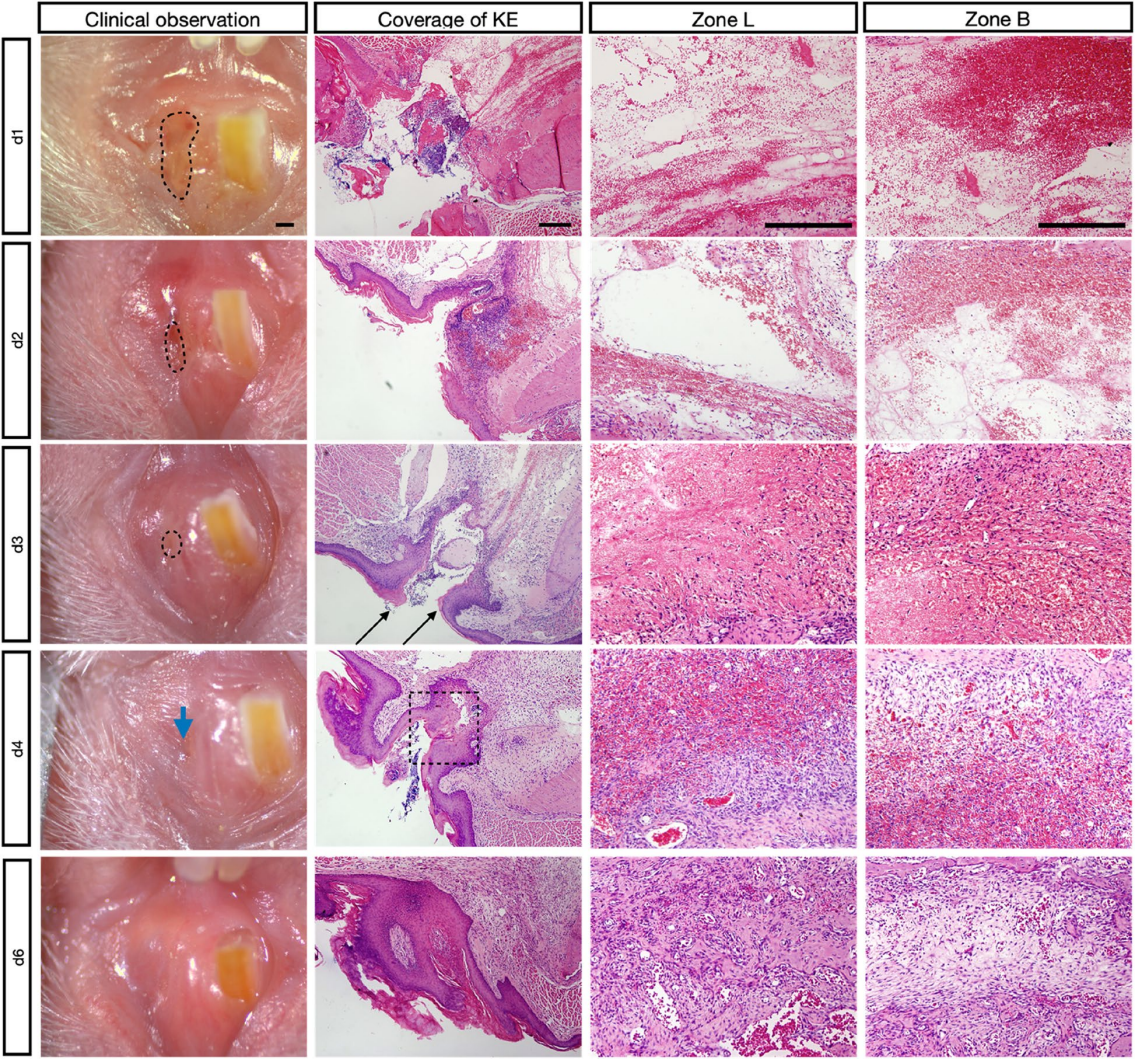
Clinical observation and histological analysis of socket at different points during the healing period with HE staining. KE at different stages, observed clinically and histologically. The black dotted lines mark the boundary between the GT or coagulum and the KE. With centripetal growth of the KE, the black line separating the GT or coagulum from the KE faded away. On d3, fusion of the migrating KE (black arrow) is nearly complete. The point of KE fusion on day 4 is indicated by the blue arrow; this point is indicated in histological views by a dotted square line. Scale bar = 200 μm

A sequential healing process was revealed by histological analyses of all sites (Fig. 2). The extraction socket was found to be mostly filled with clots, inflammatory cells and collagen fibers at d1. As the migrating epithelium recovered, a boundary of fibrous coagulum separated the KE from the remaining areas on clinical observation.

The coagulum and void were gradually replaced by mesenchymal stem cells and fibroblasts after 2 days of healing (Fig. 2). Cells with osteogenic potential and vascular structures stained with Runx2 and CD34 were individually located in lingual bone marrow (Supplement 1-B, C). Besides, we could also detect CD34 positive vascular structures in the buccal bone close to the coronal side (Fig. 5-B Black arrows). Although both clinical and histological aspects demonstrated centripetal KE growth, the sockets were still largely covered by GT accompanied by many newly formed vessels (Supplement 1-A).

At d3, PM, which consisted of many mesenchymal stem cells and vessels, gradually began to replace clots. The epithelium migrated to the central part and replaced GT. There was some space covered by GT, where no KE could be detected. Although the boundary of migrating KE became blurred, it could still be distinguished on clinical observation.

By d4, PM dominated the socket, including osteoblasts (Fig. 4 A3-E3; A4-E4), mesenchymal stem cells, fibroblasts, vessels and some woven bone (WB). Zone L contained many more cells and collagen fibers. Both clinical observation and histological analysis showed that KE reached fusion in the central region, where no GT could be detected.

By d6, Zone L was flooded with WB, accompanied by multinuclear osteoclasts located on the WB. Zone B was mainly occupied by a great amount of PM. Meanwhile, some WB formed along the surface of the buccal bone and extended toward the center.

### Description of bone formation

**Fig. 3:**
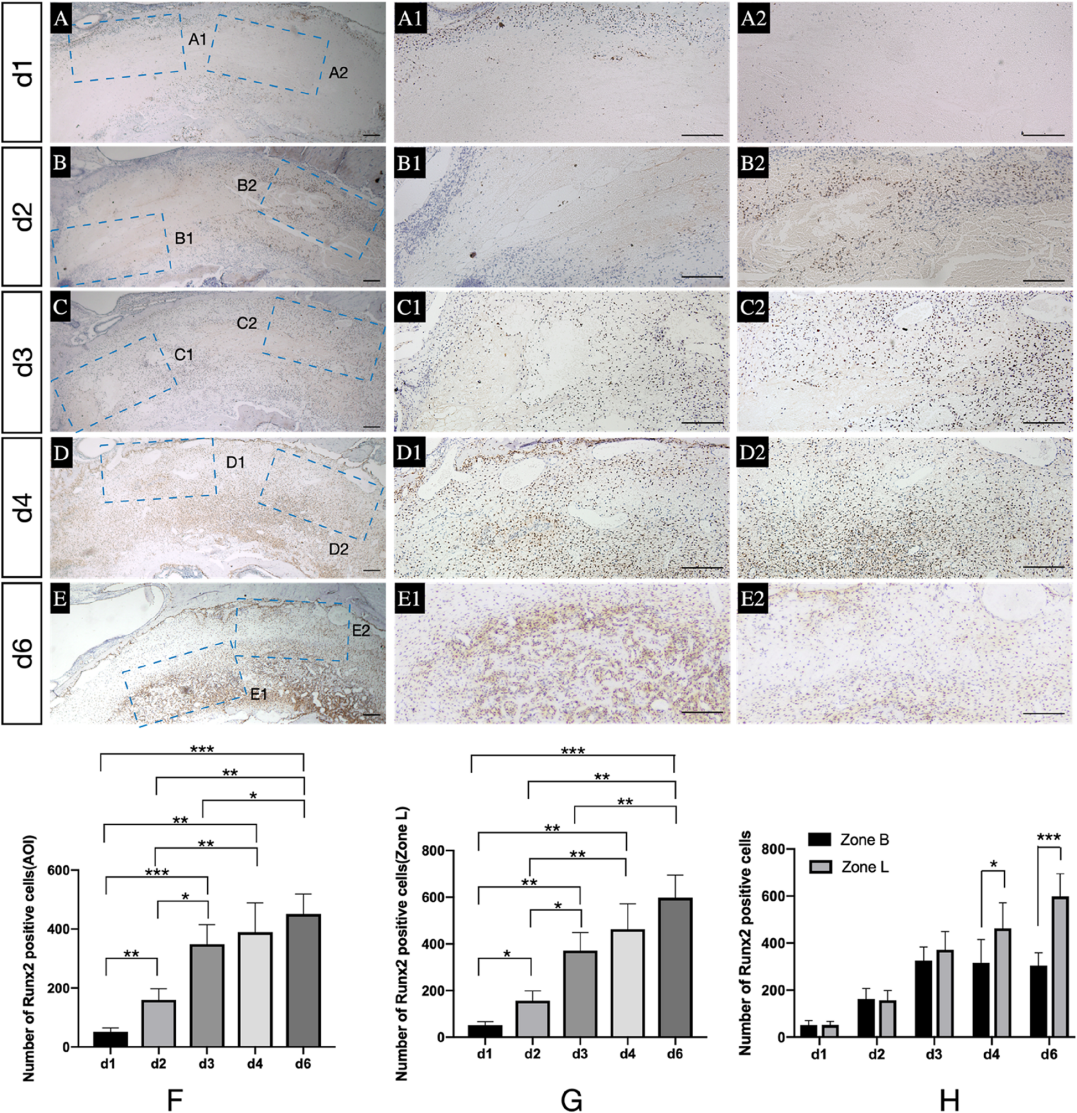
Potential for bone formation in sockets. **A**-**E** Immunohistochemical images of Runx2 expression in AOI at different healing points; A1-E1, A2-E2. The magnified picture of the blue dotted area located in the AOI individually; **F** and **G** Number of Runx2-positive cells in the AOI and Zone L at different stages; **H** Comparison of Runx2-positive cells between Zone L and Zone B. *, *p* < 0.05, **, *p* < 0.01, ***, *p* < 0.001. Scale bar = 200 μm

The expression of OC and Runx2 (Fig. 3 A-E) after tooth extraction was used to identify osteogenic potential and ability during the healing process.

The expression of Runx2 (Fig. 3 A1-E1, A2-E2, F, G) in the AOI and Zone L continued to increase and peaked on d6 compared to d1, d2 and d3 (AOI: *p = 0.0001, p = 0.002* and *p = 0.0134;* Zone L*: p = 0.0001, p = 0.0011* and *p = 0.0025*), but no significant difference could be detected when that on d4 was compare with d3(AOI: *p = 0.9117*; Zone L: *p = 0,4584*) and d6 (AOI: *p = 0.7381*; Zone L: *p = 0,3215*). And d4 had larger Runx2 positive cells than d1 and d2 (AOI: *p = 0.002, p = 0.0089*; Zone L: *p = 0.0011, p = 0.0057*).

In addition, during the same healing period, Runx2 expression in Zone L and Zone B was also compared (Fig. 3-H). On d4 and d6, Zone L had a higher number of Runx2-stained cells than Zone B (*p = 0.0341* and *p = 0.0002*).

**Fig. 4:**
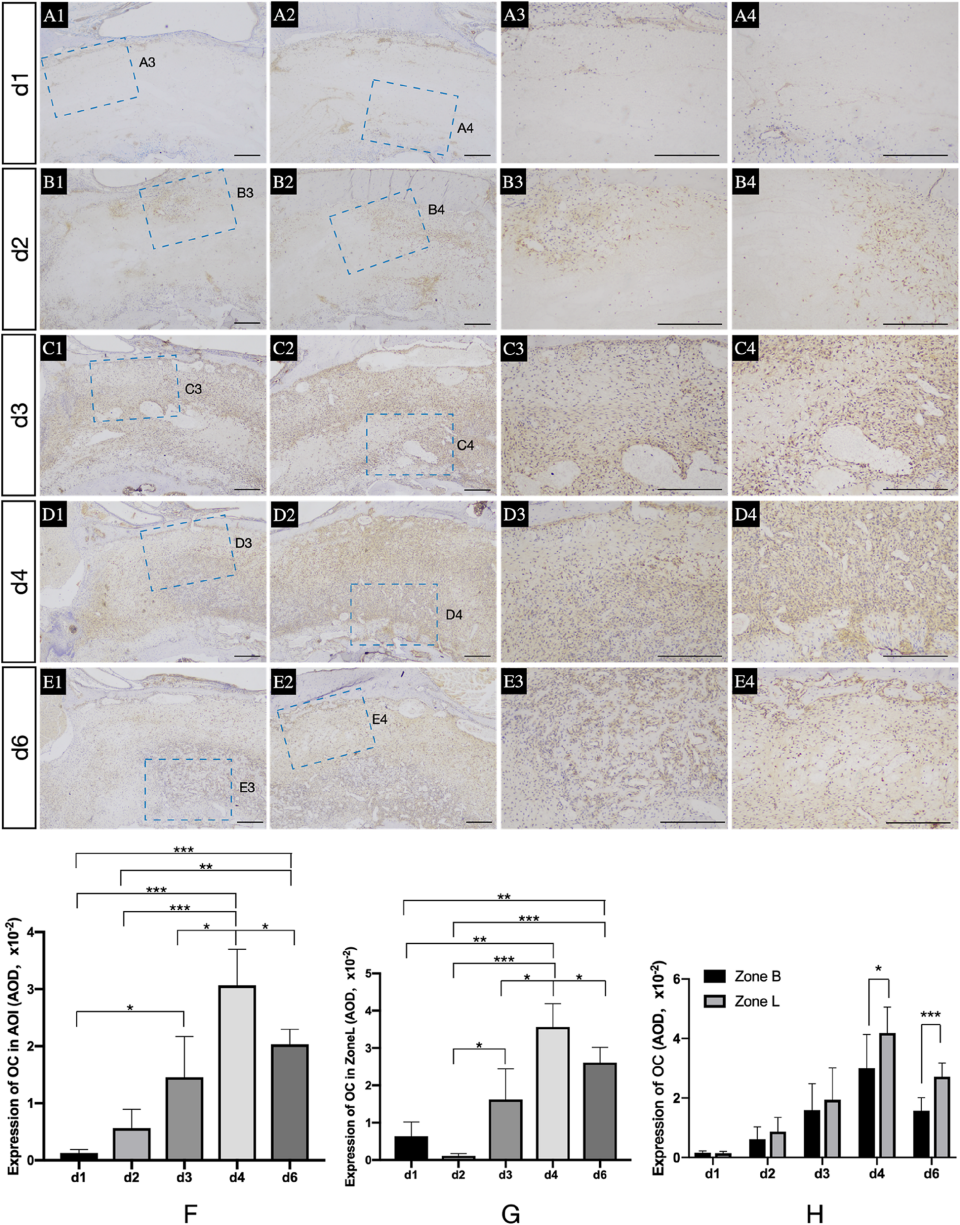
Osteoblastic bone formation in sockets. A1-E1, A2-E2. Expression of OC in coronal and middle part of AOI at different healing points individually; A3-E3. The magnified picture of the blue dotted area located in the A1-E1; A4-E4. The magnified picture of the blue dotted area located in the A2-E2; **F** and **G** Expression of OC in the AOI and Zone L at different points during the healing period. **H** Comparison of OC expression between Zone L and Zone B. *, *p* < 0.05, **, *p* < 0.01, ***, *p* < 0.001. Scale bar = 200 μm

OC was applied to evaluate the bioactivity of mature osteoblasts (Fig. 4 A1-E1, A2-E2, A3-E3, A4-E4). The OC expression in AOI and Zone L (Fig. 4- F, G) peaked on d4 during the healing period, including d1, d2, d3 and d6 (AOI: *p = 0.0002, p = 0.0009, p = 0.0321* and *p = 0.0483*) Zone L: *p = 0.001, p = 0.0001, p = 0.0293* and *p = 0.0187*). In addition, OC expression was greater in Zone L than in Zone B on both d4 and d6 (Fig. 4-H) (*p = 0.0319* and *p = 0.0004*).

### Description of newly formed vessels

**Fig. 5:**
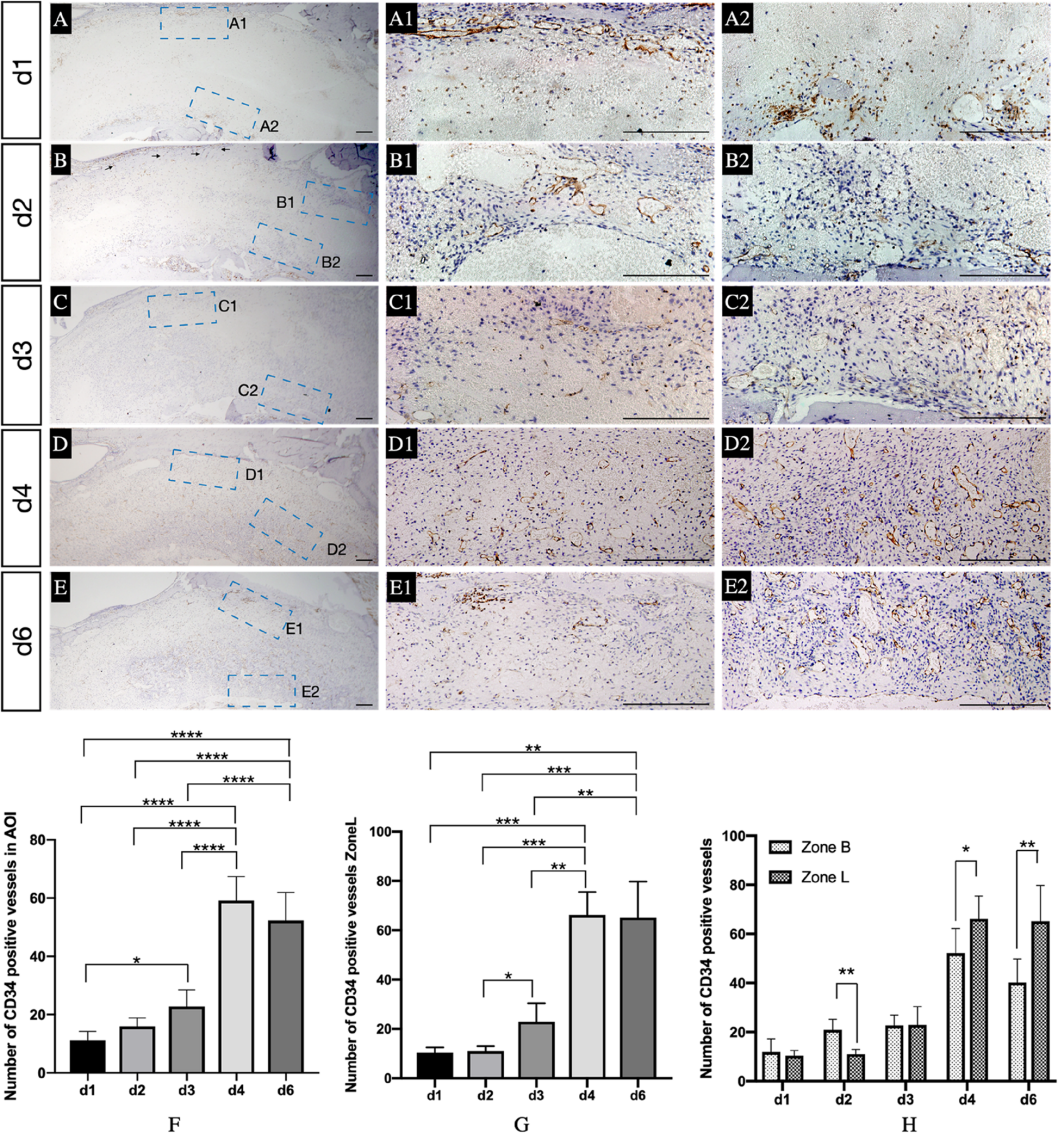
Angiogenesis in sockets. **A**-**E** Immunohistochemical images of CD34 in AOI at different healing points; A1-E1, A2-E2. The magnified picture of the blue dotted area located in the AOI individually; **F** and **G** Number of newly formed vessels in the AOI and Zone L at different points during the healing period; **C** Comparison of the number of newly formed vascular structures between Zone L and Zone B; Black arrows located in B indicated the CD34 positive vessels in the buccal bone. *, *p* < 0.05, **, *p* < 0.01, ***, *p* < 0.001, ****, *p* < 0.0001. Scale bar = 200 μm

CD34 antibody was used to identify newly formed vascular structures (Fig. 5 A-E, A1-E1, A2-E2). On d4, the number of CD34-positive vessels in the AOI and Zone L (Fig. 5-F, G) peaked compared to that on d1, d2 and d3 (AOI: *p < 0.0001, p < 0.0001* and *p < 0.0001*; Zone L: *p = 0.0001, p = 0.0002* and *p = 0.0029*). No difference could be found between d4 and d6 in either the AOI or Zone L (AOI: *p = 0.3710*; Zone L: *p > 0.9999*).

On d2, CD34 expression was greater in Zone B (Fig. 5-H) than Zone L (*p = 0.0012*). However, on d4 and d6, CD34 expression was greater in Zone L than Zone B (*p = 0.0308* and *p = 0.0071*).

### Description of osteoclast activity

**Fig. 6:**
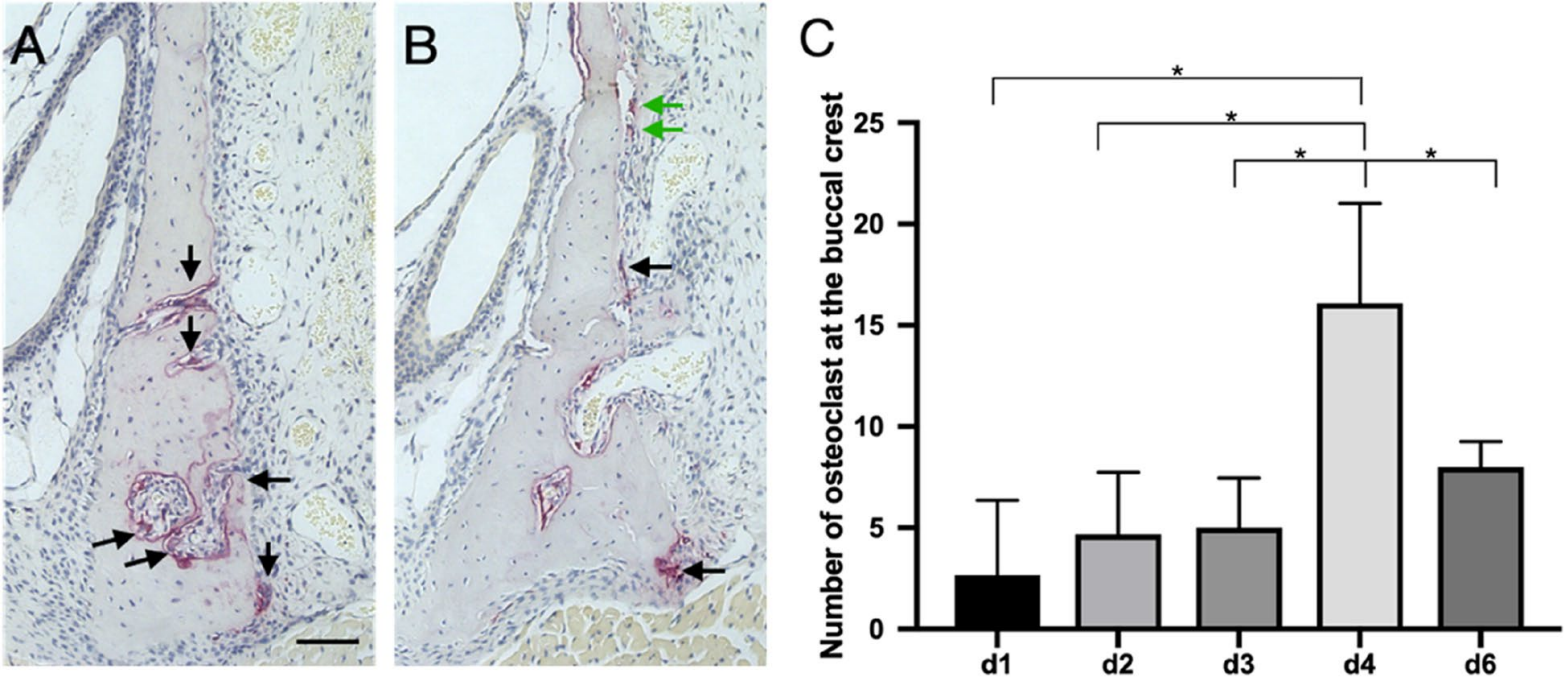
Resorption and remodeling of bone. **A** and **B** Images of TRAP-positive multinuclear osteoclasts (black arrows) located on the crest of the buccal bone at 4 d and 6 d; A few osteoclasts formed at the surface of WB (green arrows); **C** Comparison multinuclear osteoclasts on the crest of the buccal bone at different time points were detected; *, *p* < 0.05. Scale bar =100 μm

TRAP-stained multinuclear osteoclasts represent the bone resorption capacity (Fig. 6-A,B). The number of active and mature osteoclasts located along the buccal bone crest achieved its highest level on d4 when compare to d1, d2, d3 and d4(*p = 0.0218*, *p = 0.0137*, *p = 0.035* and *p = 0.0374*) during the healing period (Fig. 6-C).

### Pearson correlation coefficient

Pearson’s correlation coefficient between the area without keratinized epithelium (AWKE) and levels of immunohistochemical markers was calculated, as shown in Table 1. AKWE was significantly and negatively correlated with OC and Runx2 expression. This result showed that greater KE coverage was accompanied by tissue healing in sockets with a greater capability and potential for bone formation. CD34 and Runx2 expression were correlated with OC expression. None of the remaining markers were significantly correlated with each other (Table 1).

**Table 1:** Correlation analysis of the expression of different markers (*n* = 6)

Comparison (in the AOI)	Pearson correlation coefficient
AWKE vs. OC	r = −0.8987, *p* = 0.0354, *p* < 0.05
AWKE vs. CD34	r = −0.8420, *p* = 0.0736, NS
AWKE vs. Runx2	r = −0.9813, *p* = 0.0031, *p* < 0.01
OC vs. CD34	r = 0.948, *p* = 0.012, *p* < 0.05
Runx2 vs. CD34	r = 0.85, *p* = 0.068, NS
Runx2 vs. OC	r = 0.881, *p* = 0.049, *p* < 0.05

### Healing pattern of early implant placement

**Fig. 7:**
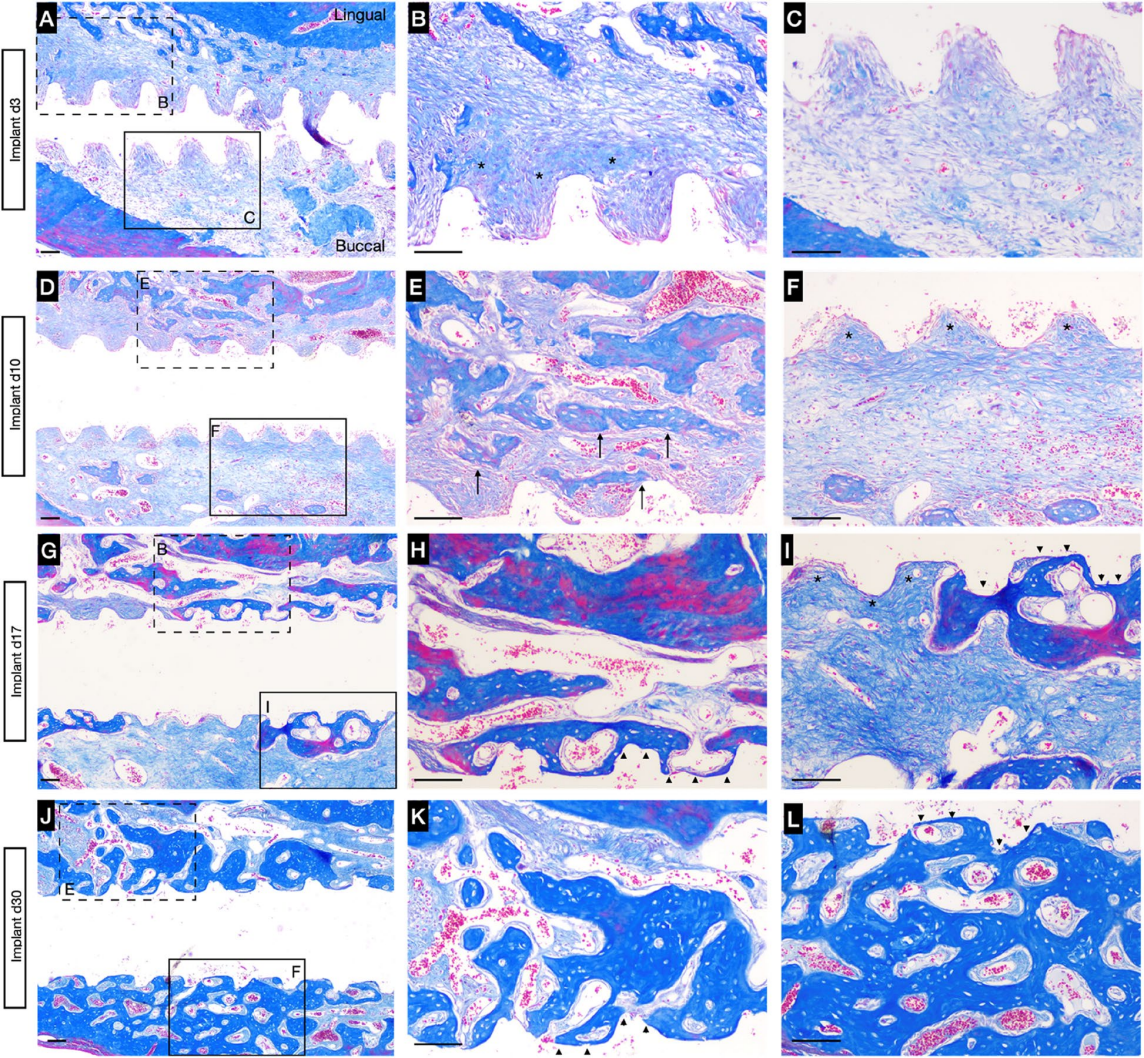
Shift in the healing process after implantation in Masson staining. Representative images of the implant at 3 d (**A**), 10 d (**D**), 17 d (**G**) and 30 d (**J**) after placement; **B**, **C**, **E**, **L**, **H**, **I**, **K** and **L** represent the high-magnification images of the boxed regions are shown for better visualization in the grafted area. Newly formed WB around the implant is indicated by black arrows. Asterisks indicate immature osteocytes around implant threads. Mature bone with bone marrow and osteocytes are indicated by arrowheads. Scale bar: 100 μm

When KE fused in the central region after 4 days’ healing of tooth extraction, an implant was inserted into the PM with the highest bioactivity. After 3 days of healing, no clots or inflammatory cells formed, and almost all threads were surrounded by collagen fibers and PM (Fig. 7. A-C, Supplement 2. A-C). In the 10 days following implant placement, more WB formed and extended from the lingual wall. In the meanwhile, only a small portion of WB could be detected in the buccal side. Both lingual and buccal surface of the implants were still occupied by dense collagen fibers (Fig. 7. D-F, Supplement 2. D-F).

After 17 days of healing (Fig. 7. G-I), both the lingual and buccal aspects of the implant were largely occupied by mature new bone. Interestingly, some PM still occupied the buccal side, and no obvious fibrous infiltration was observed (Supplement 2. G-I). The osseointegration was completed after 30 days of healing (Fig. 7. J-L, Supplement 2. J-L).

**Fig. 8:**
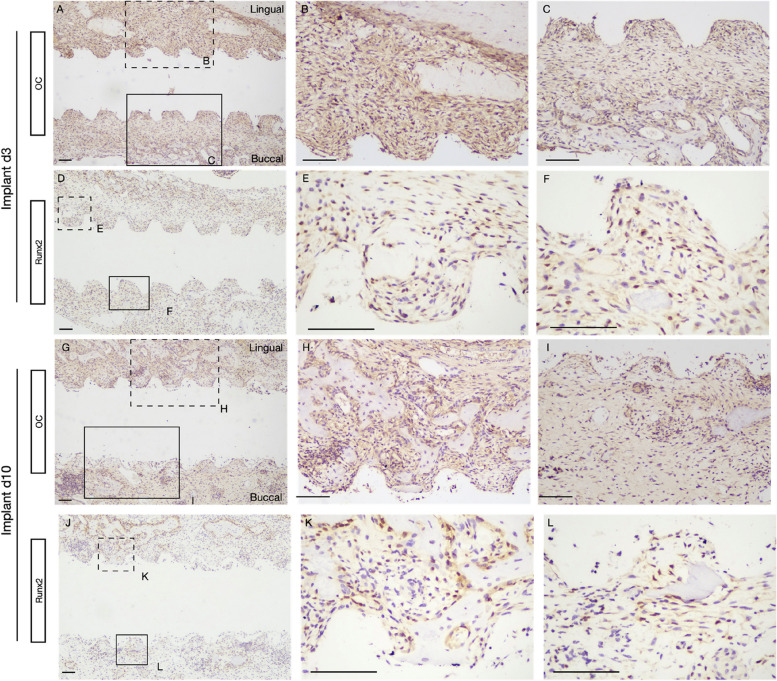
Osteoblast activity during early osteointegration. At 3 days and 10 days after implant placement, tissues were stained for OC and Runx2. Note the presence of a large amount of PM rich in mature osteoblasts and mesenchymal stem cells located around the implant surface. With the formation of bone, the number of OC-positive cells on the newly formed bone surface and around the vascular structures (arrows) gradually increased. Scale bar: 100 μm

High expression of OC and Runx2 could be both detected in tissues surrounding the surface of an implant at both 3 (Fig. 8 A-F) and 10 days (Fig. 8 G-L) after tooth extraction.

## Discussion

Despite the widespread application of early implant placement in clinical practice, the exact indication for early implantation remains unclear. This animal study first revealed that the fusion of KE could be treated as an indication of the early implant placement. Moreover, we confirm that PM in the extraction socket could maintain its osteogenic potential, beneficial for bone formation, and should be preserved carefully.

The present result confirmed that the characteristics of KE, the tissues in the socket and the number of osteoclasts located on the buccal crest from d3 to d6 were similar to those observed after approximately 3–8 weeks in clinical studies of PM domination, fast buccal bone modeling and complete KE coverage [3, 9, 12, 19].

An osteoblast activity marker, OC, acts as a calcium binding protein during bone formation and is applied to identify mature osteoblasts as a highly specific marker [20–22]. In studies of socket preservation or GBR, this marker has been widely used [23, 24]. Its accumulation in mature bone matrix laid the foundation of future bone formation and it gradually decrease with time proceed and new bone formation [25–27]. Newly formed vessels could be identified using CD34 in extraction sockets [28]. The trend of new vascular structure formation was similar to that of OC expression in this study. OC-stained cells largely enclosed vascular structures. PM obtained from healing human sockets also exhibits this phenomenon, and the primary theory for its explanation suggests that vessels contain many nutrients, growth factors and oxygen for cell differentiation and proliferation [9], which are also important for WB formation and bone remodeling [29]. Endothelial cells also contribute to the differentiation of osteoblasts [30]. Runx2 is a member of the runt domain family of transcription factors and is used to identify mesenchymal stem cells with the potential to differentiate into osteoprogenitor cells [20, 31]. It has been widely applied by many authors to identify potential osteogenesis in sockets after extraction [24, 32, 33]. There was a discrepancy between peaking time points of Runx2 positive staining and early bone formation related cytokines like Alp, Bmps and Col1a1 in the previous study or OC in this study [15]. In addition of delayed response of Bmp signaling, Runx2 also expressed in future osteoblasts. The accumulation of osteoblasts during the bone formation would accompany with upregulation of expression of Runx2 [34]. These markers presented showed when the KE fuses on the center of the alveolar socket, the PM has the highest osteogenic and angiogenic activity, which is more conducive to promoting new bone formation compared to other periods.

KE coverage was significantly correlated with the formation of PM with high bioactivity. A similar phenomenon could be also detected in dogs’ model, extraction sockets were covered by epithelium at 2 weeks; at the same time, PM occupied almost all of the sockets, but the correlation between KE coverage and PM formation was not analysed [10, 35, 36]. Some authors believe that, as a protective barrier against bacteria, KE and GT serve as the basis for future osteogenesis. The problem with GT, however, is that it also impairs bone formation [13]. Protective membranes, including native collagen membranes, nonabsorbable membranes and connective tissue grafts, can allow better effect in alveolar ridge preservation (ARP) [37–40]. Research on ARP in dogs revealed that faster PM and new bone formation and good dimensional ridge preservation could be achieved when a membrane was applied [41]. The soft biological membrane materials cannot provide good space maintenance ability. One of the main reasons was that the protection provided by the membrane could largely prevent bacterial infiltration, creating an undisturbed environment for PM and new bone formation. Rapid PM formation could allow space for tissue regeneration and prevent the ingrowth of fibrous tissue into the bone defect, as indicated by studies on the application of PM for dogs’ periodontal defects and mice’s anterior chambers [10, 11, 42]. These articles highlight the importance of the barrier function provided by artificial membranes or autologous KE. As shown in the present research, KE fusion represented the initial event in the establishment of an undisturbed environment and fully displacement of GT, and lay foundation of bone formation.

The healing pattern of the incisor socket were observed. The bioactivity of PM in Zone L was higher than that in Zone B on d4 and d6, resulting in quicker bone formation in Zone L. Interestingly, at d2, Zone B contained more CD34 positive vessels than Zone L. The buccal bone was thin but intact. Under the early action of osteoclasts, the bone marrows and blood vessels inside were quickly exposed to the socket, participating in the formation of new blood vessels on Zone B (Fig. 5-B Black arrows). The lingual side had a denser bone plate, which might take longer to expose the bone marrow inside, leading to the delayed formation of new blood vessels. After the full exposure of lingual naive bone walls, its bone marrows contained more stem cells. And their penetration became the main source of PM formation and future osteogenesis and angiogenesis (Supplement 1). This kind of osteogenesis is similar to that in healing after GBR [43, 44]. Thus, bone formation begins and is oriented by how much bone marrow is present in bony walls [45]. In clinical practice, the volume of vital bone formation is determined by the anatomy of the alveolar structures [46]. The centripetal pattern of socket healing observed in previous studies, in which thick alveolar walls mostly surrounded the socket, can also be attributed to this phenomenon [13, 35, 47]. As a result of the special anatomy of sockets investigated in this study, the osteogenic ability of PM was different, and the bone formation rate on the buccal side was different from that on the lingual side of the socket.

Then, an implant was inserted into the PM when KE fusion was achieved. In previous research on osseointegration in healed ridges of mice, osteointegration begins with clots, followed by GT formation, PM formation, WB occupation and finally mature bone modeling at about 3, 7, 14 and 21 days after implant placement individually [48, 49]. In our study, the healing process skipped the step of clot formation and the tissue surrounding the implant was PM and mature bone with bone marrows after 3 and 17 days’ healing. The difference in the healing process of osteointegration might be explained by the presence of PM, which was the first biological element in the early implant placement. At this moment, it could not be taken for granted that PM with great number of collagen fibers would be replaced by WB [9]. We used immunohistochemistry to detect the expression of OC and Runx2, which would not occur in fibrous tissue, to verify whether the tissue at these stages were PM with osteogenic potential or fibrous tissue. Finally, almost all threads were surrounded by mature bone and large portion of bone marrows at 30 days’ period. Our result indicated that the osteogenic activity of PM was not changed by the surgical procedure. And its preservation would lead to skipping the biological process of clot formation. Although this study could not confirm whether the direct contact of PM can accelerate the speed of osseointegration for lacking of control group of staged implant placement, we proved that the PM could be preserved during the surgical procedure. We first found that the bone volume after incisor extraction can accommodate longer implants than that after molar extraction, which is a good model for implant-related research. However, the differences in implant sites and anatomical structures cannot be ignored.

We also found that tissue located on the lingual side of the implant had a higher bone formation rate, which was similar to the healing pattern observed after tooth extraction. There are two possible reasons for this. One is that the lingual bone contained more bone marrow and contributed to bone formation, as we previously discussed. The second is that PM bioactivity was higher on the lingual side than on the buccal side. In conclusion, a thicker lingual bony wall and PM with greater self-regenerative ability both contribute to faster bone formation on the lingual surface of the implant.

There are some obvious advantages to this method for determining the opportunity for early implant placement. First, it is simple to identify the healing stage in which PM had high bioactivity by the clinical observation of KE. Moreover, the presence of a large amount of KE can allow primary wound closure to be achieved as quickly as possible. Thirdly, both the PM in Zone L and Zone B can achieve good osseointegration when in contact with the surface of implants, which provides important pre-clinical evidence for how to handle PM in the procedure of the early implant placement. This is also a topic that has not been discussed and researched by previous scholars.

There are some limitations to this research. The remaining apical papilla located in the apical region during the extraction process and thin bone walls in the apical region were completely different from what would be observed in a patient. Therefore, we excluded the apical region from our statistical analysis. This animal model could not completely simulate the early healing pattern in humans. Although this work can provide a relatively precise and individual indication for early implant placement, further in-depth research of large animals is needed to confirm that this indication could be applied in other clinical situations, such as the treatment of apical cysts and active or chronic infections and to confirm whether early implantation can achieve faster osseointegration.

## Conclusion

Within the limitations of this animal study, we can conclude that the migration of KE is correlated with the formation of highly osteogenic and angiogenic PM. And the fusion of KE could be treated as an indication for early implant placement. At this moment, PM contacting with the surface of implant would finally lead to perfect osseointegration.

### Supplementary Information

**Additional file 1.**

## Data Availability

All data generated or analysed during this study are included in this published article [and its supplementary information files].
