# Supplementary material for: The fusion of keratinized epithelium, an indication of early implant placement in the aesthetic area: an animal study
Source: BMC Oral Health. 2023 Dec 19;23:1016. doi: 10.1186/s12903-023-03755-9 (PMC10729501; doi:10.1186/s12903-023-03755-9)
Supplement: Supplementary file 1 — Additional file 1. [file 12903_2023_3755_MOESM1_ESM.docx]

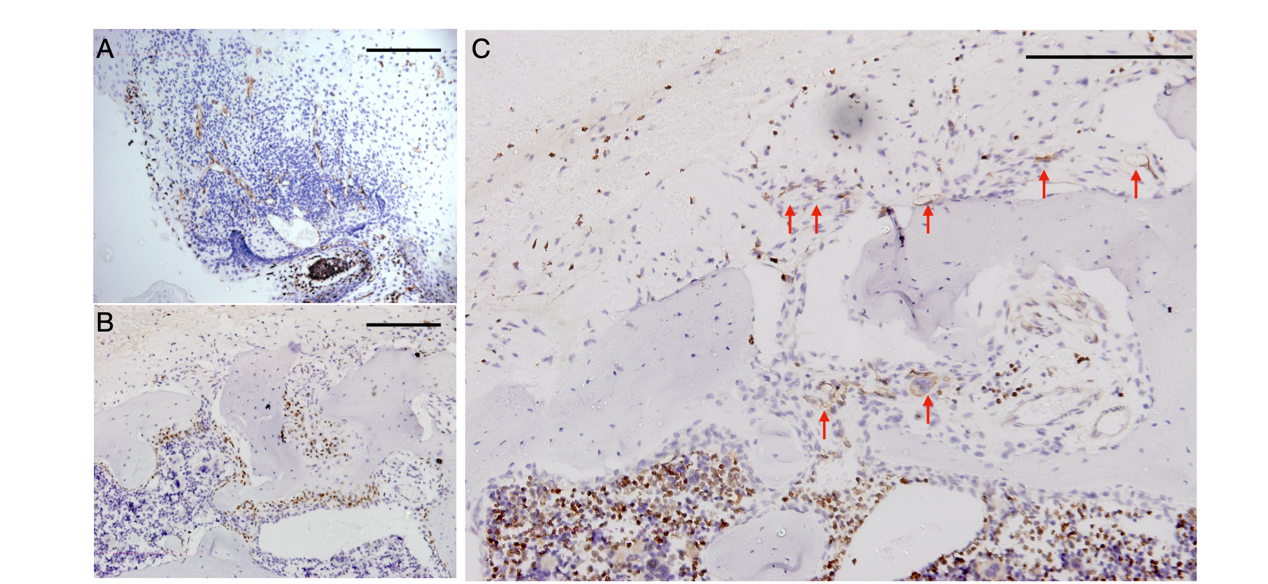


Supplement 1. Immunohistochemical localization of CD34 and Runx2 at 2 days after healing.

A, At the central part of GT, large newly formed vessels stained with CD34 could be found. B, Cells in bone marrow stained with Runx2 lined on the naïve bone and connected to the healing tissue in sockets. C, The red arrows indicated the newly formed vessels; cells stained with CD34 was located in bone marrow; and some newly formed vessels originated from bone marrow migrant toward the center of socket. Scale bar = 200 μm.


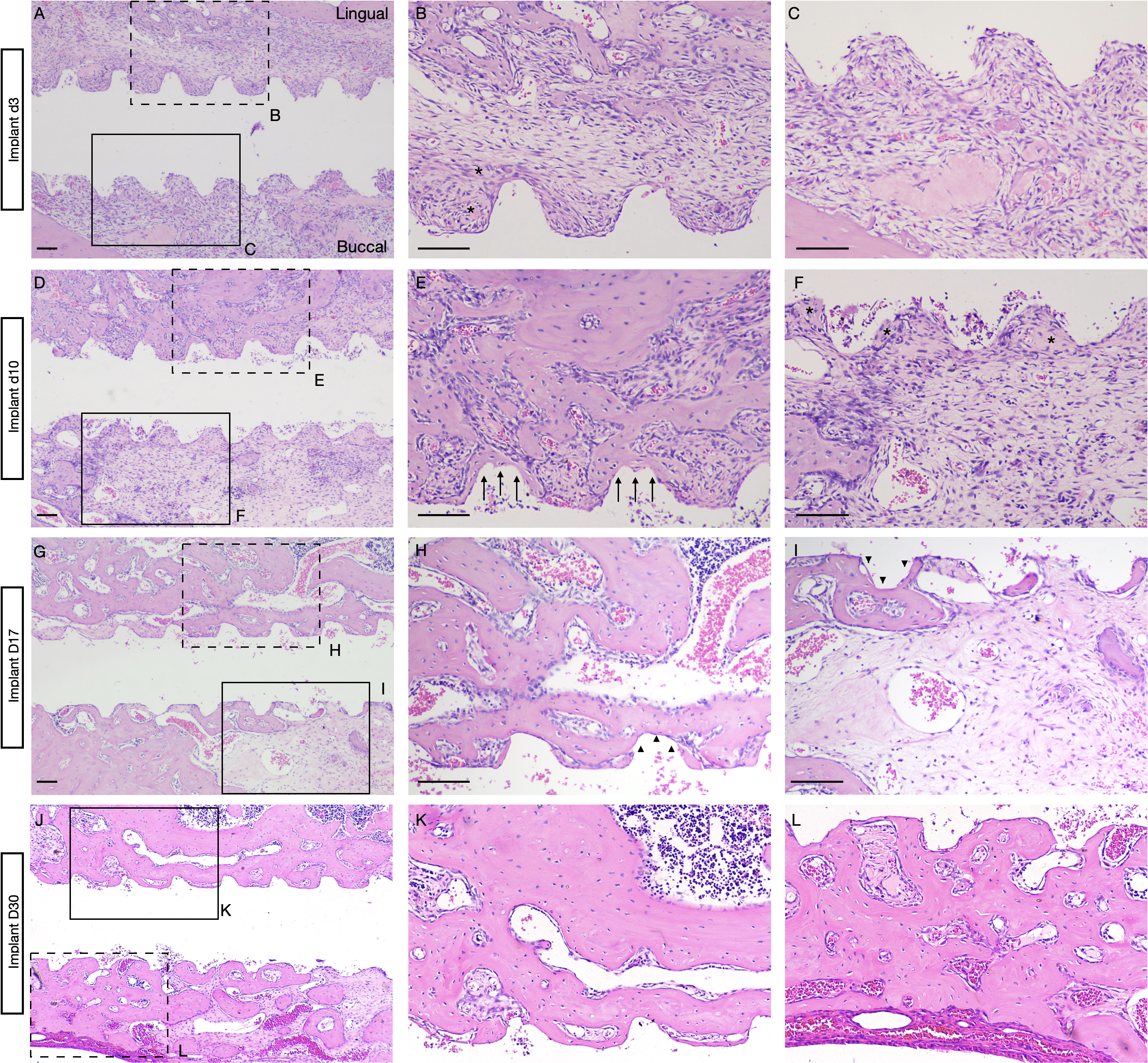


Supplement 2. Shift in the healing process after implantation in HE staining.

Representative images of the implant at 3 d (A), 10 d (D), 17 d (G) and 30 d (J) after placement; high-magnification images of the boxed regions are shown for better visualization of bone morphology in the grafted area. WB around the threads is presented by black arrows. Asterisks indicate bone matrix around the implant. Mature bone with bone marrow and osteocytes are indicated by arrowheads. Scale bar: 100 μm.


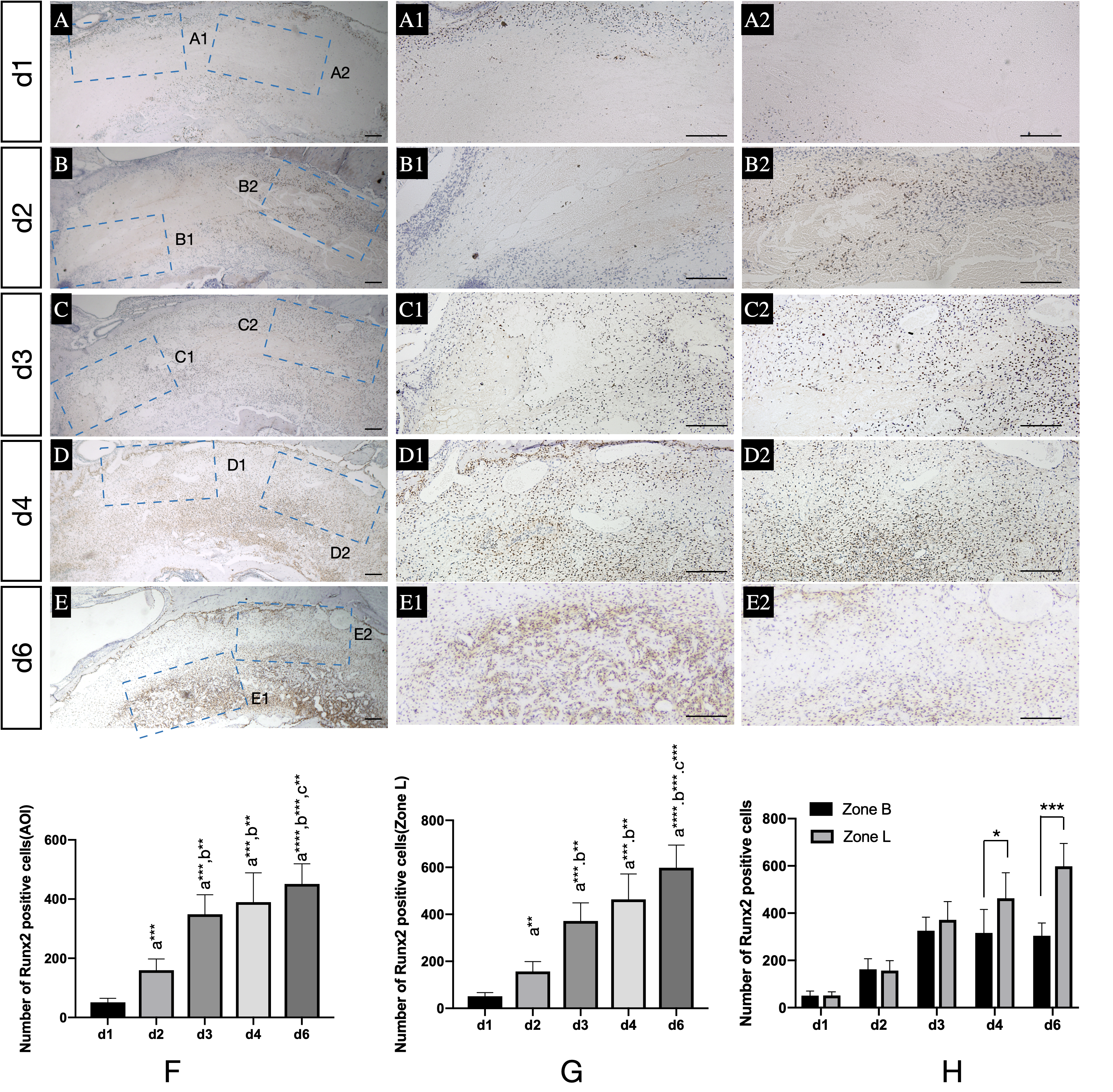


Supplement 2. Potential for bone formation in sockets.

A-E2. Immunohistochemical images of Runx2 expression in sockets at different points during the healing period. F and G. Number of Runx2-positive cells in the AOI and Zone L at different stages. H. Comparison of Runx2-positive cells between Zone L and Zone B. a: p<0.05 vs. d1; b: p<0.05 vs. d2; c: p<0.05 vs. d3; d: p<0.05 vs. d4. *, p<0.05, **, p<0.01, ***, p<0.001, ****, p<0.0001. Scale bar = 200 μm.


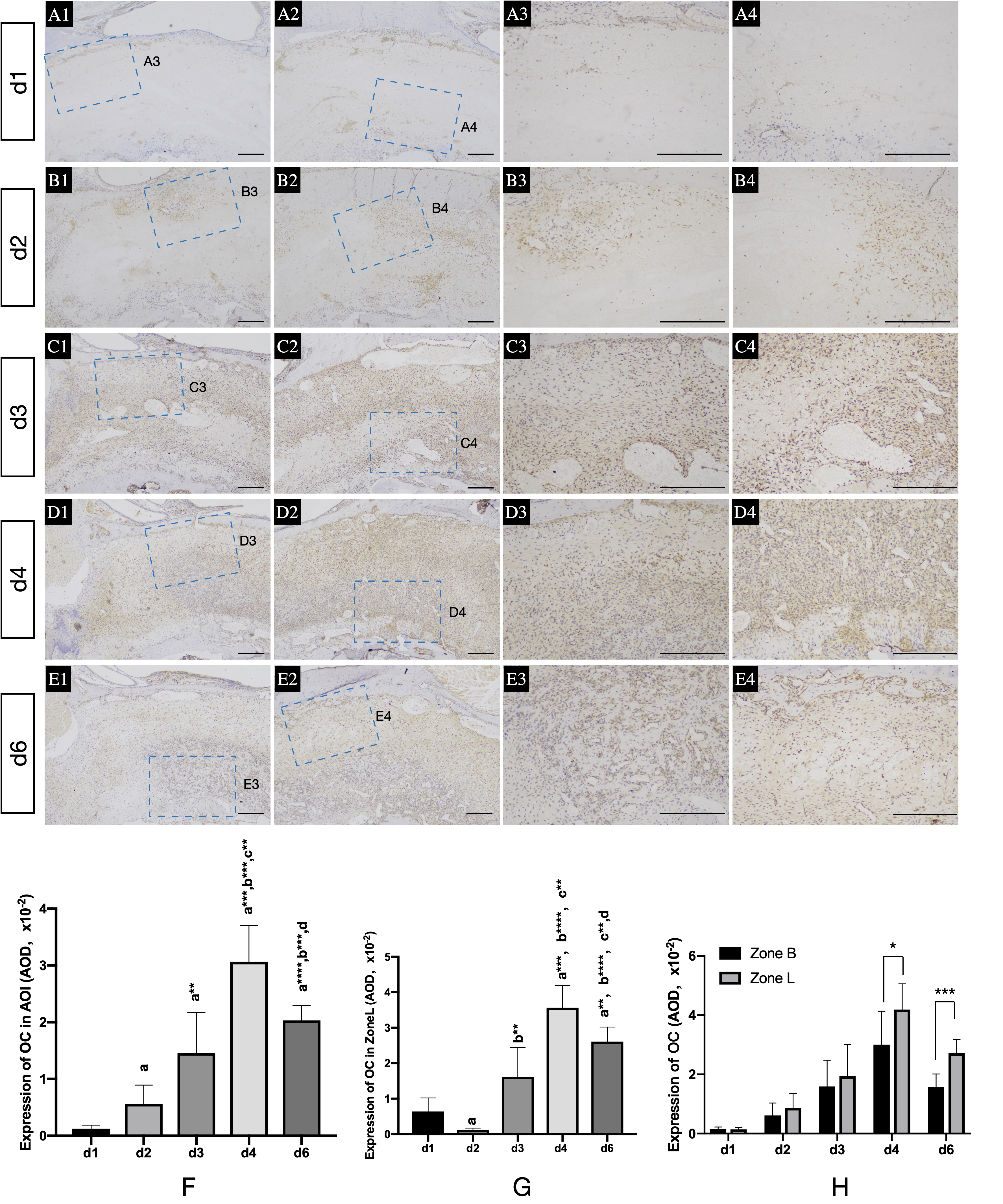


Supplement 3. Osteoblastic bone formation in sockets.

A1, B1, C1, D1 and E1. Coronal part of healing sockets. A2, B2, C2, D2 and E2. Middle part of healing sockets. All samples were stained for OC at different points during the healing period; the blue dotted line indicates magnified areas. F and G. Expression of OC in the AOI and Zone L at different points during the healing period. H. Comparison of OC expression between Zone L and Zone B. a: p<0.05 vs. d1; b: p<0.05 vs. d2; c: p<0.05 vs. d3; d: p<0.05 vs. d4. *, p<0.05, **, p<0.01, ***, p<0.001, ****, p<0.0001. Scale bar = 200 μm.


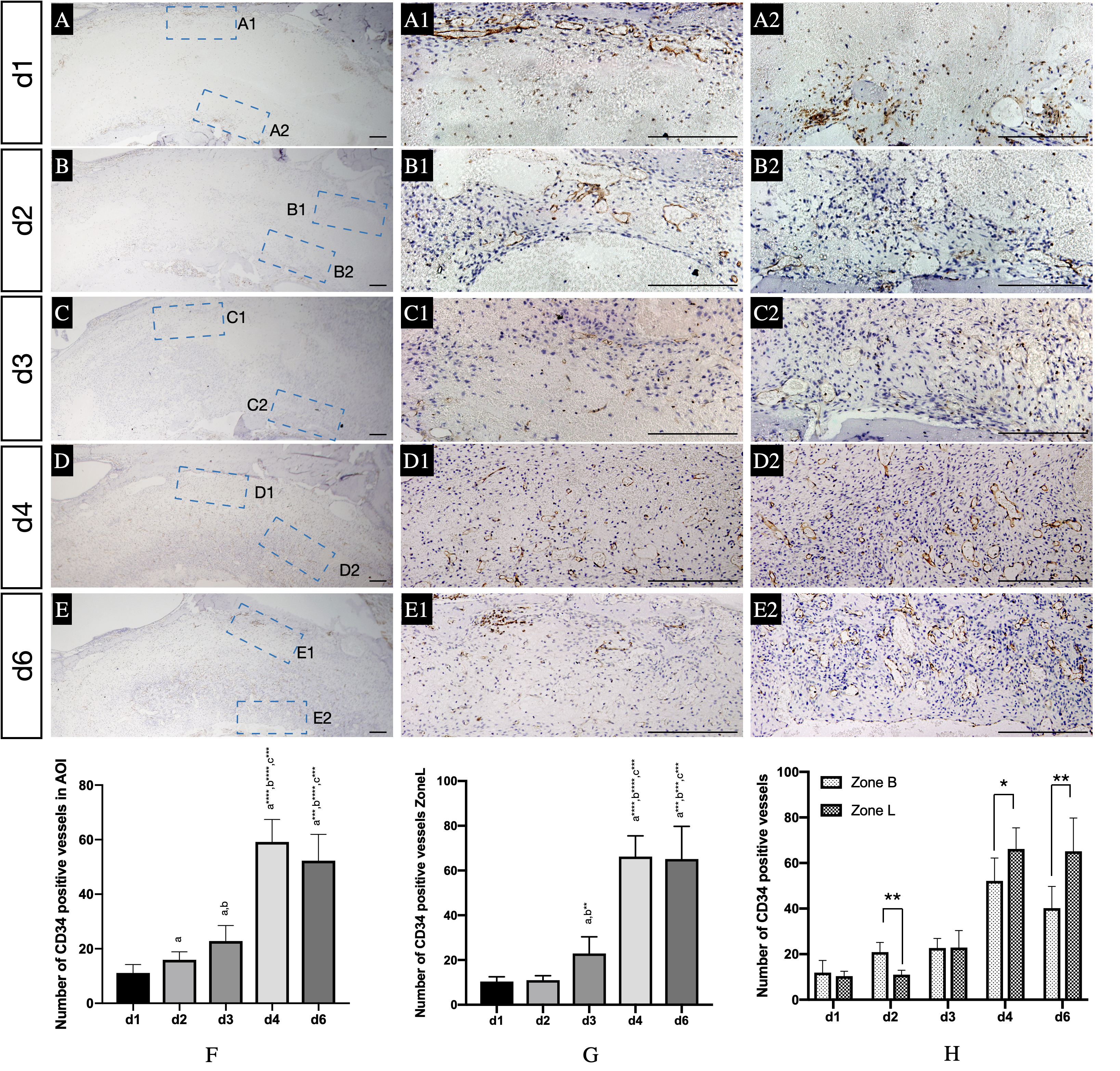


Supplement 4. Angiogenesis in sockets.

A-E2. Immunohistochemical images of CD34-positive newly formed vascular structures in areas at different points during the healing period; the blue dotted line represents magnified areas. F and G. Number of newly formed vessels in the AOI and Zone L at different points during the healing period. C. Comparison of the number of newly formed vascular structures between Zone L and Zone B. a: p<0.05 vs. d1; b: p<0.05 vs. d2; c: p<0.05 vs. d3; d: p<0.05 vs. d4. *, p<0.05, **, p<0.01, ***, p<0.001, ****, p<0.0001. Scale bar = 200 μm.


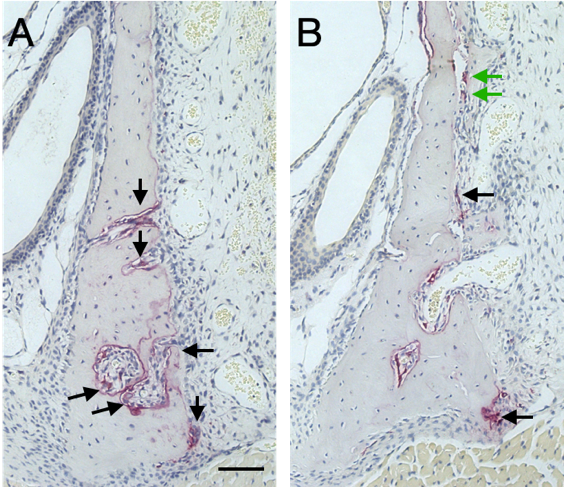


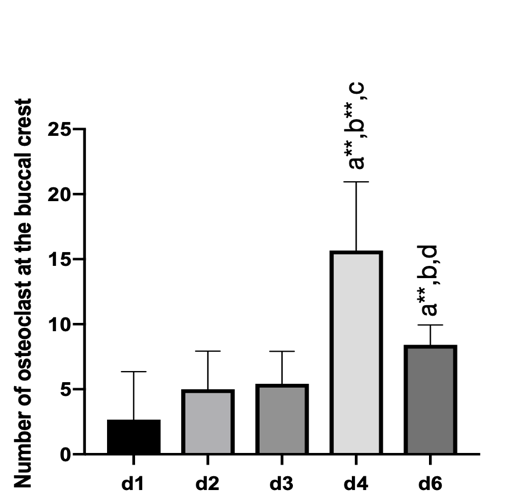


Supplement 5. Resorption and remodeling of bone.

A and B. Images of TRAP-positive multinuclear osteoclasts (black arrows) located on the crest of the buccal bone at 4 d and 6 d. A few osteoclasts formed at the surface of WB (green arrows). Scale bar =100 μm.
